# Rapid Molecular Detection of Zika Virus in Acute-Phase Urine Samples Using the Recombinase Polymerase Amplification Assay

**DOI:** 10.1371/currents.outbreaks.a7f1db2c7d66c3fc0ea0a774305d319e

**Published:** 2017-01-25

**Authors:** Ahmed Abd El Wahed, Sabri Saeed Sanabani, Oumar Faye, Rodrigo Pessôa, João Veras Patriota, Ricardo Rodrigues Giorgi, Pranav Patel, Susanne Böhlken-Fascher, Olfert Landt, Matthias Niedrig, Paolo Marinho de Andrade Zanotto, Claus Peter Czerny, Amadou A. Sall, Manfred Weidmann

**Affiliations:** Division of Microbiology and Animal Hygiene, Institute of Veterinary Medicine, Department of Animal Sciences, Georg-August-University, Goettingen, Lower Saxony, Germany; Hospital das Clínicas, School of Medicine, University of São Paulo, São Paulo, Brazil; Arbovirus and viral hemorragic fever unit, Institut Pasteur de Dakar, Dakar, Senegal; Hospital das Clínicas, School of Medicine, University of São Paulo, São Paulo, Brazil; Municipal Hospital of Tuparetama, Tuparetama, Pernambuco, Brazil.; University of Santo Amaro, São Paulo, São Paulo, Brazil; TIB MOLBIOL Syntheselabor GmbH, Berlin, Germany; Georg-August-University Goettingen; TIB MOLBIOL Syntheselabor GmbH, Berlin, Germany; Robert Koch InstitutRobert Koch Institut; Laboratory of Molecular Evolution and Bioinformatics, Department of Microbiology, Biomedical Sciences Institute, University of São Paulo, São Paulo, Brazil; Division of Microbiology and Animal Hygiene, Institute of Veterinary Medicine, Department of Animal Sciences, Georg-August-University, Goettingen, Lower Saxony, Germany; Arbovirus and viral hemorragic fever unit, Institut Pasteur de Dakar, Dakar, Senegal

**Keywords:** diagnostics, point of need, zika virus

## Abstract

Background: Currently the detection of Zika virus (ZIKV) in patient samples is done by real-time RT-PCR. Samples collected from rural area are sent to highly equipped laboratories for screening. A rapid point-of-care test is needed to detect the virus, especially at low resource settings.

Methodology/Principal Findings: In this report, we describe the development of a reverse transcription isothermal recombinase polymerase amplification (RT-RPA) assay for the identification of ZIKV. RT-RPA assay was portable, sensitive (21 RNA molecules), and rapid (3-15 minutes). No cross-reactivity was detected to other flaviviruses, alphaviruses and arboviruses. Compared to real-time RT-PCR, the diagnostic sensitivity was 92%, while the specificity was 100%.

Conclusions/Significance: The developed assay is a promising platform for rapid point of need detection of ZIKV in low resource settings and elsewhere (e.g. during mass gathering).

## Introduction

Zika virus (ZIKV) associated with severe congenital malformations (e.g., microcephaly, hydrocephalus) and neuropathies (e.g. Guillian-Barré-like syndrome) has been declared a public health emergency of International concern by WHO 1 ^,^ 2. In the absence of a specific treatment and vaccine, early diagnostics is key to control the epidemic and trigger intervention. Diagnostic challenges are unspecific symptoms in the context of co-circulating Dengue virus and Chikungunya virus, limited laboratory infrastructure in rural areas where most cases occur, cross reactivity impeding serological detection and need for rapid detection. Here we report on the development and use of a simple, mobile, point of need molecular assay based on reverse transcription recombinase polymerase amplification (RT-RPA) assay for detection ZIKV in urine in 15 minutes.

## Methods

**Ethics statement**

Human urine samples tested in Brazil were provided by the Laboratory of Medical Investigation in Dermatology and Immunodeficiency, LIM- 56/03, São Paulo Institute of Tropical Medicine, Faculty of Medicine the University of São Paulo, São Paulo, Brazil, which has obtained the ethical approval from the Faculty of Medicine, São Paulo University Review Board (FMUSP N0 1.184.947). All study participants provided informed consent.

**Test development**

In order to determine the analytical sensitivity of the RT-RPA assay, ZIKV NS1/NS2 molecular RNA standard was ordered at concentration of 1e10/µl from GenExpress (Gesellschaft für Proteindesign mbH, Berlin, Germany). The RT-RPA primers and probe (FP: 5´-TCTCTTGGAGTGCTTGTGATTCTACTCATGGT-3´; RP, 5´-GCTTGGCCAGGTCACTCATTGAAAATCCTC-3´; exo-probe, 5´-CCAGCACTGCCATTGA(BHQ1-dT)(Tetrahydrofuran)(FAM-dT)GCTYATDATGATCTTTGTGGTCATTCTCTTC-phosphate-3´) were designed in NS2A region conserved among all ZIKV lineages (nt 3572 to 3713, GeneBank: LC002520.1). The European Network for Diagnostics of Imported Viral Diseases (ENIVD) provided flaviviruses, alphaviruses and arboviruses that were used in the testing the cross-reactivity of the ZIKV RT-RPA assay. The clinical performance of the assay was evaluated on acute-phase (2-10 days of onset of symptoms) urine samples collected from suspected cases at the Municipal Hospital of Tuparetama, Pernambuco, Brazil. The nucleic acid extraction and RT-RPA assay were applied as previously described 3 and all results were compared with a real-time RT-PCR 4 as gold standard.

**Statistical methods**

A semi-log regression analysis and a probit analysis were performed by plotting the RT-RPA threshold time (Tt) against the number of molecules detected to determine the ZIKV RT-RPA assay analytical sensitivity using PRISM (Graphpad Software Inc., San Diego, California) and STATISTICA (StatSoft, Hamburg, Germany), respectively. Diagnostic sensitivity and specificity were calculated using standard formulas. In addition, a linear regression analysis was performed using the values of real-time RT-PCR cycle threshold (Ct) and RT-RPA Tt by PRISM.

## Results

The limit of the detection of the assay was 21 RNA copies/reaction (95% probit analysis of dataset of eight RT-RPA runs using NS1/NS2 RNA diluted standard, Analytical sensitivity of ZIKV RT-RPA assay Fig 1 and 2). The RT-RPA assay identified African (GenBank: AY632535) and Brazilian strains (Instituto Evandro Chagas, Belém, Brazil) down to 65 and 35 RNA genome equivalents, respectively, using ten-fold serial dilutions from virus culture supernatant.

**Analytical sensitivity of ZIKV RT-RPA assay.**
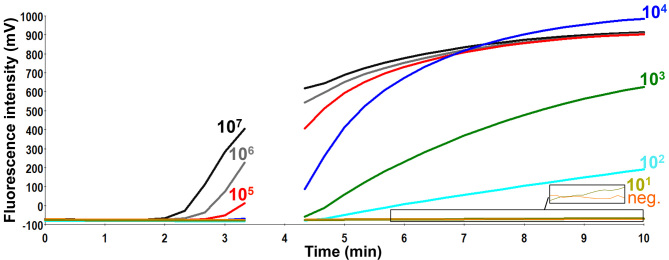
Fluorescence development via real-time detection in one RT-RPA run by using a dilution range of 1e7-1e1/µl of the RNA molecular standards (Graph generated by ESEquant tubescanner studio software). The limit of detection was 10 RNA copies. Data of eight RT-RPA runs is used for the probit regression analysis in Fig 2. The box in the lower right corner of the figure magnifies the fluorescence signals for the ten RNA copies and the negative control as the signal for ten RNA copies is very low.

**The probit regression analysis using data of eight RT-RPA assay runs.**
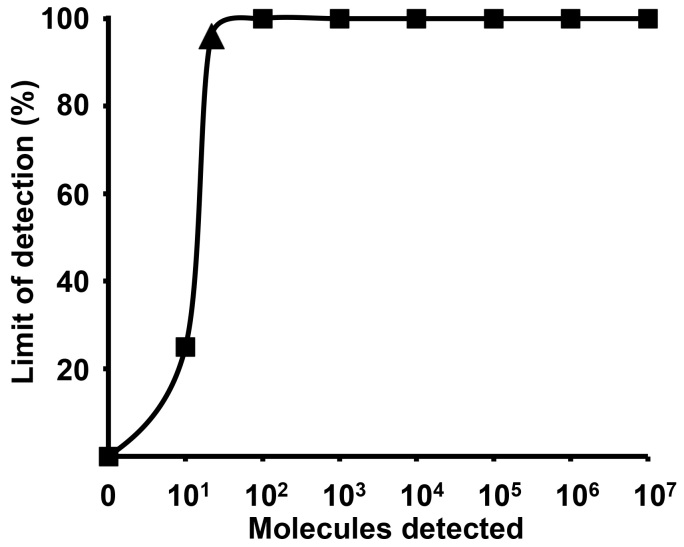
The limit of detection at 95% probability (21 RNA molecules/reaction, confidence interval 15-36) is depicted by a triangle. 1e7-1e2 RNA molecules were detected 8 out of 8 runs, while 1e1 copies were identified 2 out of 8 runs of the ZIKV RT-RPA assay.

The assay is highly specific as no amplification was observed with the following viruses: Dengue 1-4, West Nile, Yellow Fever, Tick borne encephalitis, Japanese Encephalitis, Rift Valley Fever and Chikungunya. The clinical performance of the RT-RPA assay was tested using 25 positive (Ct values: 30-39, Fig 3) and nine negative urine samples collected during the ZIKV epidemic in Tuparetama, Brazil. The RT-RPA identified 23/25 (Sensitivity: 92%) positive (Fig 3) and 9/9 (specificity: 100%) negative samples.

**Results of screening 25 urine samples with both real-time RT-PCR and RT-RPA assays.**
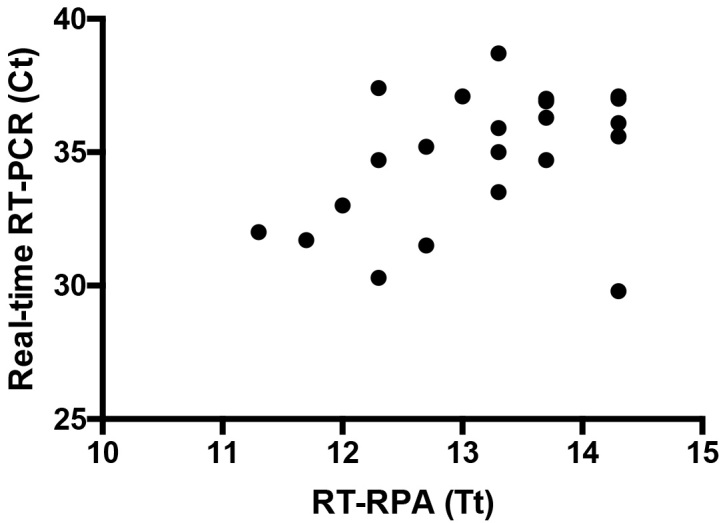
Twenty samples are shown as three samples produced identical results and two were negative in RT-RPA assay. Linear regression analysis of real-time RT-PCR cycle threshold values (Ct, Y-axis) and RT-RPA threshold time in minutes (TT, X-axis) were determined by PRISM. No correlation was found between TT and Ct values (R2 = 0.17) since the RT-RPA is much faster than the real-time RT-PCR even with samples with high Ct value.

## Conclusions

To our knowledge, the developed RT-RPA assay is the first sensitive rapid molecular assay applied on field samples for the detection of ZIKV in 15 minutes, which could be implemented at the point of need in a mobile suitcase laboratory 5 to make testing of pregnant women available directly in rural settings. Moreover, combining it with the developed dengue 6, chikungunya 7 and Sigma 8 RT-RPA assays will allow its use during outbreak investigations. Sigma virus infects exclusively dipterans, which is a good candidate to be used in the future as an extraction and RPA reaction control.

## Data Availability Statement

All data underlying the findings described in the manuscript is fully available in the body of the manuscript without any restriction.

## Conflict of Interest Statement

All authors except Pranav Patel and Olfert Landt are in the public research sector. Mentioned authors are employed by Tib MolBiol, a manufacturer of oligonucleotides. This does not alter the authors´ adherence to all the scientific policies on sharing data and materials.

## Corresponding Author

Dr. Ahmed Abd El Wahed, Email: abdelwahed@gwdg.de and abdelwahed@me.com
